# Reactive Oxygen Species and NRF2 Signaling, Friends or Foes in Cancer?

**DOI:** 10.3390/biom13020353

**Published:** 2023-02-11

**Authors:** Ruolei Wang, Lirong Liang, Misaki Matsumoto, Kazumi Iwata, Atsushi Umemura, Feng He

**Affiliations:** 1 The Center for Cancer Research, Academy of Integrative Medicine, Shanghai University of Traditional Chinese Medicine, Shanghai 201203, China; 2 Department of Respiratory and Critical Care Medicine, Beijing Institute of Respiratory Medicine and Beijing Chao-Yang Hospital, Capital Medical University, Beijing 100020, China; 3 Department of Pharmacology, Kyoto Prefectural University of Medicine, Kyoto 602-8566, Japan

**Keywords:** NRF2, metabolism, oxidative stress, ROS, inflammation, unfolded protein response

## Abstract

The imbalance between reactive oxygen species (ROS) production and clearance causes oxidative stress and ROS, which play a central role in regulating cell and tissue physiology and pathology. Contingent upon concentration, ROS influence cancer development in contradictory ways, either stimulating cancer survival and growth or causing cell death. Cells developed evolutionarily conserved programs to sense and adapt redox the fluctuations to regulate ROS as either signaling molecules or toxic insults. The transcription factor nuclear factor erythroid 2-related factor 2 (NRF2)-KEAP1 system is the master regulator of cellular redox and metabolic homeostasis. NRF2 has Janus-like roles in carcinogenesis and cancer development. Short-term NRF2 activation suppresses tissue injury, inflammation, and cancer initiation. However, cancer cells often exhibit constitutive NRF2 activation due to genetic mutations or oncogenic signaling, conferring advantages for cancer cells’ survival and growth. Emerging evidence suggests that NRF2 hyperactivation, as an adaptive cancer phenotype under stressful tumor environments, regulates all hallmarks of cancer. In this review, we summarized the source of ROS, regulation of ROS signaling, and cellular sensors for ROS and oxygen (O_2_), we reviewed recent progress on the regulation of ROS generation and NRF2 signaling with a focus on the new functions of NRF2 in cancer development that reach beyond what we originally envisioned, including regulation of cancer metabolism, autophagy, macropinocytosis, unfolded protein response, proteostasis, and circadian rhythm, which, together with anti-oxidant and drug detoxification enzymes, contributes to cancer development, metastasis, and anticancer therapy resistance.

## 1. Introduction

All living organisms communicate with the environment and the environment poses constant threats to disrupt cell functions and shape its fate. A wide variety of exogenous and endogenous stressors, such as ionizing radiation, prooxidants, hypoxia, nutrition deprivation, iron deficiency, viral infection, lipid overload, metabolic stress, and proteotoxic aggregates, can lead to the production of various reactive oxygen species (ROS), including superoxide anion (O2^•−^), hydrogen peroxide (H_2_O_2_), the hydroxyl radical (^•^OH), and the hydroperoxyl radical (^•^OOH), peroxyl (RO_2_^•^), alkoxyl (RO^•^), and reactive nitrogen species (RNS), such as nitric oxide (^•^NO) and peroxynitrite (ONOO^−^) [1,2]. ROS and RNS, collectively referred to as ROS/RNS, have inherent chemical properties that confer reactivity to different biological molecules such as lipids, proteins, and nucleic acids to affect their functions, thus causing nitrosative and oxidative stress. Here, for simplicity, we refer to them as ROS. Under healthy conditions, cells and tissues in our body are exposed to greatly varying levels of O_2_, with significant differences in the partial pressure of O_2_ in distinct anatomical sites under physiological conditions [3]. An imbalance in O_2_ demand and supply as well as abnormal metabolism can lead to the generation of ROS. Low and moderate levels of ROS may contribute to normal cellular physiology as signaling molecules, whereas high levels of ROS lead to tumor development by inducing DNA mutations and oncogenic transformation [4]. Notably, cancer cells often display high levels of cellular GSH and ROS, in particular chemoresistant cancer cells, in which higher ROS levels activate antioxidant defense mechanisms, including nuclear factor erythroid 2 (NF-E2)-related factor 2 (NRF2), for the development of chemoresistance by reprogramming metabolism and alleviating drug-mediated oxidative stress that normally leads chemosensitive cancer cells to death [5].

In the balance of ROS accumulation and antioxidant defense mechanisms, organisms develop evolutionarily conserved stress response programs to restore homeostasis or adapt to the stress. When the stress is severe, prolonged, or the stress response signaling is dysregulated, it leads to cell death, tissue injury, metabolic dysfunction, and inflammation which all increase the risk for pathological disorders, particularly cancer. Timely induction and resolution of inflammation induced by tissue injury is an attempt of the organism to clear the non-reparable dead cells and pathogens, which facilitates the tissue repair and wound healing process, and ultimately brings about a stress-free state. When stress is severe or prolonged, the unresolved inflammation turns chronic and tumor-promoting, contributing to dysregulated stress response signaling and cancer hallmarks. The oxidative stress response is one of the most important stress response programs. Sensing oxidative stress and orchestrating different signaling pathways enable the affected tissues or cells to cope with the stressors or to restore homeostasis which has strong implications in tumorigenesis, cancer prevention, and cancer treatment. In this review, we first summarized the sources of ROS, regulation of ROS signaling, and cellular sensors for ROS and O_2_, including the recent progress on the regulation of ROS generation and the master regulator of the anti-oxidative stress response, NRF2 signaling. We focused on the new functions of NRF2 in cancer development, including regulation of cancer metabolism, autophagy, macropinocytosis, unfolded protein response, proteostasis, and circadian rhythm, which, together with its canonical anti-oxidant and drug detoxification enzymes, contributes to cancer development, metastasis, and cancer drug resistance. Our knowledge of the ROS and NRF2 signaling provides critical insights into mechanisms of cancer formation and new cancer treatment opportunities for targeting NRF2 signaling in cancer.

## 2. Source of ROS and Regulation of ROS Generation

The appearance of atmospheric O_2_ into the biosphere was one of the defining events in evolution, as it allowed the development of highly efficient energy production from oxidative phosphorylation and substrate utilization for the production of cellular constituents, which shaped the evolutionary development of aerobic life forms. Oxidation and reduction are two of the most important processes in metabolism. An imbalance in O_2_ demand and supply leads to the production of ROS and the resulting oxidative stress. Although the concentration of ambient atmospheric O_2_ is approximately 21%, normally defined as normoxia, physiologic O_2_ concentrations in our body are dependent on the partial pressure of oxygen (pO_2_) in the system with an average of approximately 5% (physoxia), but it varies greatly among organs, usually ranging from 3% to 10% [6]. O_2_ levels in lung alveoli reach as high as 14.5% and decrease further in arterial blood and peripheral tissues [3]. In some tissues, such as the kidney medulla and bone marrow, O_2_ levels are even lower and around 1.3–3% [7]. In addition, O_2_ levels often fluctuate dynamically under different physiological and pathological conditions. For instance, in normal pregnancy, the extravillous cytotrophoblast cells occlude the uterine spiral arterioles creating a physiological low O_2_ environment in the first trimester, and this is essential for pregnancy success, but when it occurs later in pregnancy, it is pathological and associated with common pregnancy complications, such as preeclampsia, where O_2_ concentrations can be around 3% [8,9]. O_2_ levels in most tumors are much lower than the respective normal tissue, ranging from 0.3–4.2% with an average of approximately 1% [3]. Therefore, cancer cells grow under the typical condition of pathological hypoxia (<1% O_2_ level). Fluctuated O_2_ supply and demand, as well as cellular metabolism, contribute to ROS and they play important roles in the regulation of cell signaling, such as proliferation, differentiation, and oncogenic transformation. ROS and antioxidant defense are two sides of a coin in play. Cancer cells usually display high ROS levels, although they are equipped with sufficient antioxidant defense machinery. Some malignancies display higher ROS levels than others, for instance, melanoma, which has the highest ROS levels. Melanoma must face additional ROS sources that do not affect other cancers, including UV radiation and the melanin production process that contributes to ROS generation [10]. Therefore, ROS levels and antioxidant defense signaling, such as NRF2, could have important implications for targeted cancer treatment.

### 2.1. ROS Produced from Mitochondrial Metabolism

**Figure 1:**
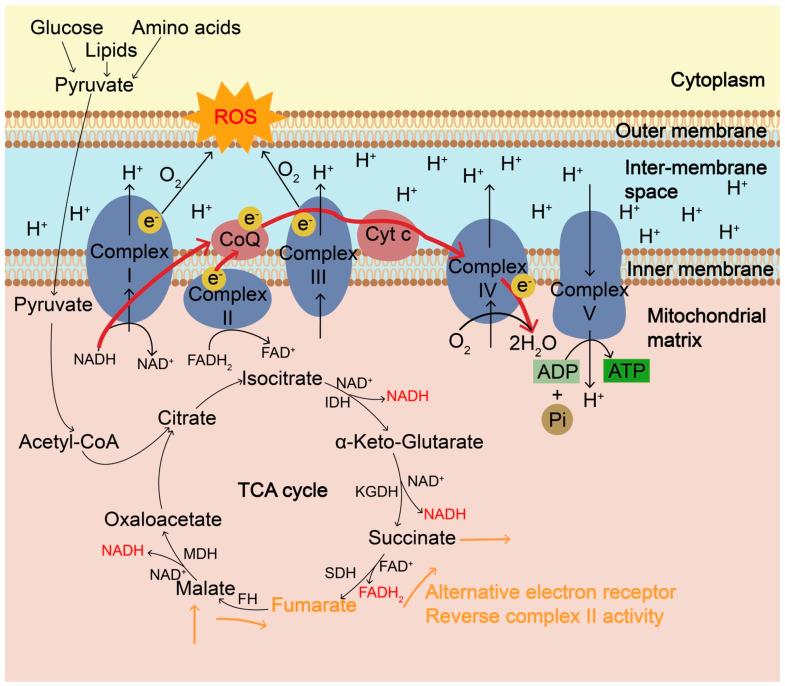
The mitochondrial electron transport chain (ETC) and the generation of ROS. Mitochondrial ETC, coupled with tricarboxylic acid (TCA) cycle, is the main cellular source of ROS in aerobic organisms and ROS are mainly formed as byproducts of oxidative phosphorylation during ATP synthesis. ETC is composed of four multiprotein complexes, Complex I–IV. Complex I and ubiquinone of complex III are the major sites of ROS generation. During the TCA cycle, NADH produced from isocitrate dehydrogenase (IDH), α-ketoglutarate dehydrogenase (KGDH) and malate dehydrogenase (MDH) goes to the ETC and electrons transfer to O_2_ through Complex I and complex III. During hypoxia or high O_2_ demand, such as oncogene activation and nutrient excess, partial one-electron reduction of O_2_ can occur at complexes I and III, producing O2^•−^ due to electron leakage, resulting in the accumulation of ROS. ROS is also accumulated when complex IV is inhibited and electrons cannot be transferred effectively to O_2_. In addition to the ultimate electron acceptor O_2_, during hypoxia, fumarate can be a terminal electron acceptor and can accept electrons via the reverse complex II activity, during which fumarate is reversibly converted into succinate catalyzed by succinate dehydrogenase (SDH), accompanied by the conversion of FADH2 into FAD. Fumarate can shuttle reducing power through reversible conversions among malate, fumarate, and succinate catalyzed by fumarate hydratase (FH) and SDH, especially from an O_2_-poor tissue to an O_2_-rich one. ROS, reactive oxygen species; ETC, electron transport chain; TCA, tricarboxylic acid; IDH, isocitrate dehydrogenase; KGDH, α-ketoglutarate dehydrogenase; MDH, malate dehydrogenase.

The most common intracellular source of ROS is mitochondrial metabolism. Energy metabolism in the mitochondria, which depends on nutrient flux and cellular metabolic needs, is associated with the production of ROS. During nutrient flux and oxidation, electrons are transferred between substrates during redox reactions and continuously enter and flow through the mitochondrial electron transport chain (ETC) (Figure 1). The ETC consists of mainly four complexes containing redox centers that normally facilitate the transfer of electrons to their final acceptor O_2_. Nutrients, such as glucose, lipids, and amino acids, are taken up and metabolized inside the cells and enter the tricarboxylic acid (TCA) cycle or Krebs cycle in the mitochondrial matrix for further oxidization and energy production. The resulting electrons from the oxidative reaction are transferred to electron carriers such as nicotinamide adenine dinucleotide (NAD^+^) and flavin adenine dinucleotide (FAD) to produce NADH and FADH2, respectively. These electrons in NADH and FADH2 are then donated to the electron transport chain (ETC) at complexes I and II, respectively, where electrons are further shuttled to complex III via ubiquinone and subsequently conveyed to complex IV via cytochrome c. Four electrons are finally donated to O_2_ to generate two molecules of H_2_O. During hypoxia or high O_2_ demand such as oncogene activation and nutrient excess, partial one-electron reduction of oxygen can occur at complexes I and III, producing O2^•−^ due to electron leakage, which causes ROS accumulation, or reversed electron transport [11]. The relative ROS contributions of mitochondrial sites vary significantly and also depend on different substrates. In addition to the typical ROS signaling cascades originating from ETC and TCA cycle, recent studies revealed that reduced O_2_ reduction and hypoxic environments drive the succinate dehydrogenase (SDH) complex in reverse to use fumarate to accept electrons instead of O_2_ in the TCA cycle [12,13]. Under those conditions, fumarate is a terminal electron acceptor and can shuttle reducing power through reversible conversions among malate, fumarate, and succinate, especially from an O_2_-poor tissue to an O_2_-rich one. Malate is transported from O_2_-rich tissues into O_2_-poor tissues, where it is reversibly converted into fumarate catalyzed by fumarate hydratase (FH). Fumarate can accept electrons and be reversibly converted into succinate catalyzed by succinate dehydrogenase (SDH), accompanied by the conversion of FADH2 into FAD. The fumarate reduction leads to the accumulation of succinate and supports complex II reversal (Figure 1). Accumulated succinate can be transported out of the cell and taken up by O_2_-rich tissues for energy usage. During environmental stress (hypoxia, toxins, etc.), coenzyme Q (CoQ)-reducing enzymes are often inhibited and it leads to increased levels of reduced CoQH2. Increased levels of reduced CoQH2 and fumarate are the prerequisite for complex II to work in reverse, which participates in the sulfide metabolism for the clearance of toxic H_2_S [14]. Of note, fumarate reduction is much less efficient than succinate oxidation [15]. It is unclear whether this reaction sufficiently supports the reverse activity of complex II in mitochondria in vivo and how effectively to limit ROS accumulation.

### 2.2. ROS Produced from Nicotinamide Adenine Dinucleotide Phosphate (NADPH) Oxidases

**Figure 2:**
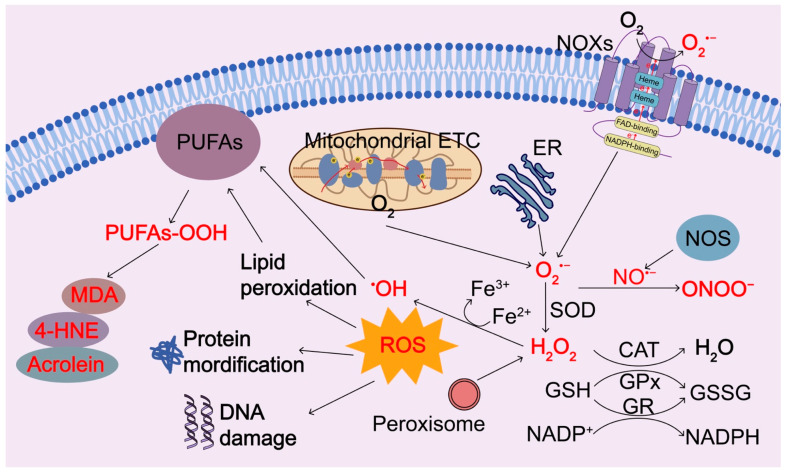
Regulation of ROS generation and clearance. Intracellular ROS are primarily produced by electron leak from aerobic respiration in mitochondria, oxidation of NADPH by NADPH oxidase enzymes (NOXs), oxidation processes in the endoplasmic reticulum (ER), and peroxisomes during normal O_2_ metabolism. Superoxide (O_2_^−^) is rapidly converted into hydrogen peroxide (H_2_O_2_) by compartment-specific superoxide dismutases (SODs). H_2_O_2_ is capable of oxidizing cysteine residues on proteins to initiate redox biology. H_2_O_2_ may be converted to H_2_O by cellular antioxidant proteins, such as glutathione peroxidase (GPx) and catalase (CAT) with the concomitant conversion of glutathione to oxidized glutathione, which is then reduced by glutathione reductase (GR) at the expense of NADPH. When H_2_O_2_ levels increase uncontrollably, hydroxyl radicals (OH⋅) form via reactions with ferrous ions (Fe^2+^) and irreversibly damage cellular macromolecules. Nitric oxide synthase (NOS) can induce the production of nitric oxide (^•^NO), which interacts with O2^•−^ and forms ONOO^−^. ROS or oxidants can modify intracellular macromolecules, such as proteins, lipids, and DNA to initiate redox biology and damage the functions of these macromolecules. In particular, ROS can induce peroxidation of polyunsaturated fatty acids (PUFAs), resulting in the products of peroxidized lipids and their breakdown products 4-hydroxy-2-nonenal (4-HNE), malondialdehyde (MDA), and acrolein. 4-HNE, 4-hydroxy-2-nonenal; MDA, malondialdehyde; SOD, superoxide dismutase; GPx, glutathione peroxidase; CAT, catalase; NOXs, NADPH oxidase enzymes; ER, endoplasmic reticulum; GR, glutathione reductase; ETC, electron transport chain.

Another major source of ROS is transmembrane nicotinamide adenine dinucleotide phosphate (NADPH) oxidases (NOXs) (Figure 2). NOXs are a family of membrane-bound proteins that function to transfer electrons to the final electron receptor O_2_, resulting in the generation of O2^•−^ and other downstream ROS metabolites. NOXs family includes five NOX isoforms (NOX1, NOX2, NOX3, NOX4, and NOX5) and two related enzymes, dual oxidase 1 (DUOX1) and DUOX2, which have a similar catalytic core but distinct tissue distribution, domain structure, subunit requirements, and regulatory mechanisms [16]. Among them, NOX2 is the most extensively studied and abundantly expressed in the phagocytes such as polymorphonuclear neutrophils, eosinophils, monocytes, and macrophages, where NOX2 in phagosomes membrane generates O2^•−^, which further generates other microbicidal ROS, collectively named oxidative bursts, to kill microbial pathogens. The phagocyte NOXs and ROS production are crucial in the host′s immune defense against microbial pathogens. NOXs are multicomponent enzymes and include two integral membrane proteins, 91-kDa glycoprotein p91 phagocyte oxidase (p91phox), also called NOX2, and adaptor protein p22phox forming the catalytic core of the enzyme, and three cytoplasmic regulatory subunits p40phox, p47phox, p67Phox. The catalytic subunit of NOXs is the electron transfer chain of the active NOXs and contains a conserved cytoplasmic C-terminal dehydrogenase domain with an NADPH binding site, a FAD binding site, and a conserved transmembrane domain with two histidines that bind to two hemes (Figure 2) [17,18]. The spatial separation of NOXs components enables the enzyme to be dormant in resting cells. Upon stimulation, their cytosolic components migrate almost instantly to the membrane, where they assemble and form the active enzyme. Activated NOXs catalyze the transferring of electrons from the NADPH to FAD in the cytoplasmic side of the membrane, then pass the electrons sequentially to the transmembrane inner and outer heme group, and finally convey the electrons to O_2_ on the opposite side of the membrane, forming O2^•−^, which is quickly converted to H_2_O_2_ spontaneously or mediated by compartment-specific SOD [17,18]. In this way, NOXs transport electrons across the membrane from a cytosolic electron donor to an electron acceptor in the extracellular or lumenal space, causing oxidative bursts to kill microorganisms. H_2_O_2_ can be diffusive across the membranes [19]. NOX family members locate at different cell membranes, including the plasma membrane, nuclear membrane, and endoplasmic reticulum (ER) membrane, contribute to the compartmentalization of ROS generation. In addition, nitric oxide synthase (NOS) can induce the production of nitric oxide (^•^NO), which interacts with O2^•−^ and forms ONOO^−^. 

### 2.3. Other Sources of ROS Generation and Antioxidant Regulation

ROS are also produced in the ER and peroxisomes during autoxidation processes, such as drug detoxification, xanthine metabolism, and fatty acid oxidation (Figure 2). Generation of these ROS is mediated by compartment-specific enzymes and more than forty ROS-generating enzymes have been identified in humans [20]. ER oxidative protein folding is also a key ROS producer, which is mediated by ER chaperones and oxidoreductases. The contribution of each organelle to the total cellular ROS production varies between cell types. Mitochondria, peroxisomes, and ER communicate with each other to sense ROS accumulations and redox imbalances through trans-organellar transport and diffusion at their membrane contact sites [21]. Under favorable conditions, ROS is constantly being produced at basal levels and they are quickly scavenged by different antioxidant mechanisms, thus causing no damage. Superoxide accumulation can damage and inactivate proteins containing iron-sulfur clusters and it is rapidly converted into H_2_O_2_ spontaneously, particularly at low pH or catalyzed by superoxide dismutase (SOD) 1, SOD2, and SOD3, which are located in the cytoplasm, mitochondrial matrix, and extracellular space, respectively [22]. H_2_O_2_ can be reduced to H_2_O by catalase (CAT) or be converted into the highly reactive ^•^OH via the Fenton reaction in the presence of ferrous ion (Figure 2) [11]. Glutathione peroxidase (GPx), SODs, and CAT are the most important enzymes of the cell antioxidant defense system for eliminating ROS. CAT is an antioxidant enzyme produced in all aerobic organisms. GPx catalyzes the reduction of H_2_O_2_ and peroxide radicals to H_2_O and alcohols, respectively, as well as O_2_ via the oxidation of reduced GSH into its disulfide form (GSSG) [23]. Glutathione reductase (GR) mediates the reduction of glutathione for the GSH regeneration at the expense of NADPH, which is also a cofactor used in anabolic reactions [23]. In addition, a transmembrane protein paraoxonase 2 (PON2), which is ubiquitously expressed in most cells and tissues, has been shown to scavenge cellular ROS and prevent oxidative stress, although its mechanism of action remains unclear [24,25]. A high expression of PON2 was observed in multiple types of solid tumors, conferring their resistance to oxidative stress and chemotherapy as well as other unfavorable stress conditions [25,26]. PON2 has been considered as a molecular biomarker for the prognosis of multiple cancers and an important therapeutic target [27,28].

### 2.4. Adverse Effects of ROS Accumulation

The amount and duration of ROS challenges determine their outcomes. Among the ROS, ^•^OH is the most reactive and short-lived, and it causes damage at or near the site of formation. H_2_O_2_ is a relatively weak but more stable oxidant, which can travel across the membranes by facilitating transport via channels, such as aquaporins, or by passive diffusion throughout the cells and cellular organelles [19,29]. The reactivity of O2^•−^ is in between. Because of its electric polarity, O2^•−^ has little or no membrane permeability but can pass the membrane facilitated by anion exchange protein channels [19]. O2^•−^ is spontaneously converted to H_2_O_2_ or catalyzed by SODs to generate H_2_O_2_. H_2_O_2_ is highly selective for the reversible modification of the thiol group of cysteine residues. ROS generation and dissemination is a chain reaction that results in the production of numerous breakdown molecules. At the physiologic levels, H_2_O_2_ and NO^•−^ also function as signaling molecules, while the high reactive O_2_^•−^ and ONOO^−^ can damage intracellular macromolecules, such as proteins, lipids, and nucleic acids (Figure 2). ROS or oxidants can attack lipids that contain C-C double bonds and particularly polyunsaturated fatty acids (PUFAs), resulting in lipid peroxidation. The peroxidized lipids and their breakdown products 4-hydroxy-2-nonenal (4-HNE), malondialdehyde (MDA), and acrolein act as signaling molecules to stimulate inflammation and metabolism or toxic molecules to induce apoptosis or ferroptosis [30,31]. Notably, 4HNE is an α, β-unsaturated hydroxyalkenal, resulting from peroxidation of ω6-PUFAs and linoleic and arachidonic acid. MDA, a highly reactive and toxic three-carbon dialdehyde, is derived from the decomposition of certain peroxidized lipid products, cyclooxygenase-mediated prostaglandin breakdown, or various amino acids and carbohydrates. Acrolein is the simplest unsaturated aldehyde and the most reactive product of lipid peroxidation. Lipid peroxidation is a hallmark of ferroptosis. Uncontrolled lipid peroxidation and the production of lipid peroxyl radicals, hydroperoxides, and various oxidation products enable the conversion of signaling ROS to toxic ROS, leading to ferroptosis [31]. 

Aberrant functions of these ROS-producing enzymes and ROS levels are associated with various human diseases. Chronic granulomatous disease (CGD), characterized by a deficiency in one of the components of the NOXs, is associated with life-threatening bacterial and fungal infections due to an absence of ROS production [32]. Excessive NOX2 activation and the resulting ROS production contribute to the pathophysiology of human vascular diseases and chronic liver diseases, such as atherosclerosis, hypertension, steatohepatitis, and hepatic fibrosis. In response to arterial injury, NOX2-deficient mice showed decreased neointimal formation [33]. p47phox deletion in atherogenic *ApoE^-/-^* mice decreased the atherosclerosis lesion progression [34]. In addition, deficiency of either NOX1 or NOX4 reduced lipid peroxidation and ROS production in mice with liver fibrosis, leading to attenuated liver injury, inflammation, and fibrosis [35]. In a diethylnitrosamine (DEN)-induced mouse liver cancer model, *Nox1^-/-^* mice developed significantly fewer and smaller tumors than those of WT mice, which contributed to the NOX1 deficiency in macrophages, not hepatocytes or hepatic stellate cells [36]. NOX1 deficiency in macrophages decreased ROS accumulation and production of inflammatory cytokines that promote tumor development. Expression of NOX1 by macrophages promotes hepatic tumorigenesis by inducing the production of inflammatory cytokines and ROS. In addition, nonalcoholic fatty liver disease (NAFLD) is attracting attention as a causative disease of hepatocellular carcinoma (HCC), especially with regard to its fibrosis development. NOX1 has been shown to be involved in the progression of NAFLD [37] and the aggravation of liver fibrosis [38].

## 3. Sensors of ROS and O_2_

Excessive ROS production, designated as oxidative distress, results in molecular modifications, including proteins, lipids, and nucleic acids, leading to aberrant functions of proteins, lipids, and genes, which are the fundamental basis for the pathology of tissues and organisms. At physiological levels, ROS function as signal molecules via various reversible modifications of proteins or nucleic acids to regulate cell functions, thus referring to ROS signaling or oxidative eustress. H_2_O_2_ is the major ROS in the redox-dependent regulation of biological processes and cells maintain the physiological H_2_O_2_ under tight control in the concentration range of 1-100 nM [39]. In contrast, the intracellular concentration of O2^•−^ is much lower, in the 10^–11^ M range [39]. During evolution, aerobic species developed conserved mechanisms to sense O_2_ levels and adjust metabolism to regulate O_2_ consumption, in order to cope with conditions of insufficient O_2_ supply. At a systemic level, the carotid body, a small cluster of chemoreceptor cells and supporting sustentacular cells in the adventitia of the bifurcation of the common carotid artery, is the O_2_-sensing organ for monitoring arterial blood O_2_ levels, thereby modulating cardiovascular and respiratory function primarily through sympathetic tone [40]. The carotid sinus nerve (CSN) provides sensory innervation to the chemoreceptor tissue, which includes neuron-like type I glomus cells and glia-like type II sustentacular cells. At a molecular level, almost all cells have the capacity to sense and adapt to the endogenous and exogenous O_2_ and ROS levels [40]. Sensory receptors detecting diverse external stimuli utilize ion channels to initiate the transduction of touch and temperature, or G-protein-coupled receptors (GPCRs) to trigger the signal cascade of visual, olfactory, and taste [41]. In contrast, ROS sensing in cells is executed by different O_2_ or ROS-dependent biochemical mechanisms.

The uncontrolled proliferation of cancer cells consumes a significant amount of O_2_, causing the development of a hypoxic tumor microenvironment (TME). The hypermetabolic cancer cells utilize more O_2_ than the surrounding normal cells and constantly demand more O_2_, resulting in an inadequate O_2_ supply in the tissue and organ [42].

### 3.1. The Primary Sensor of Oxidative Stress—The NRF2-KEAP1 System

**Figure 3:**
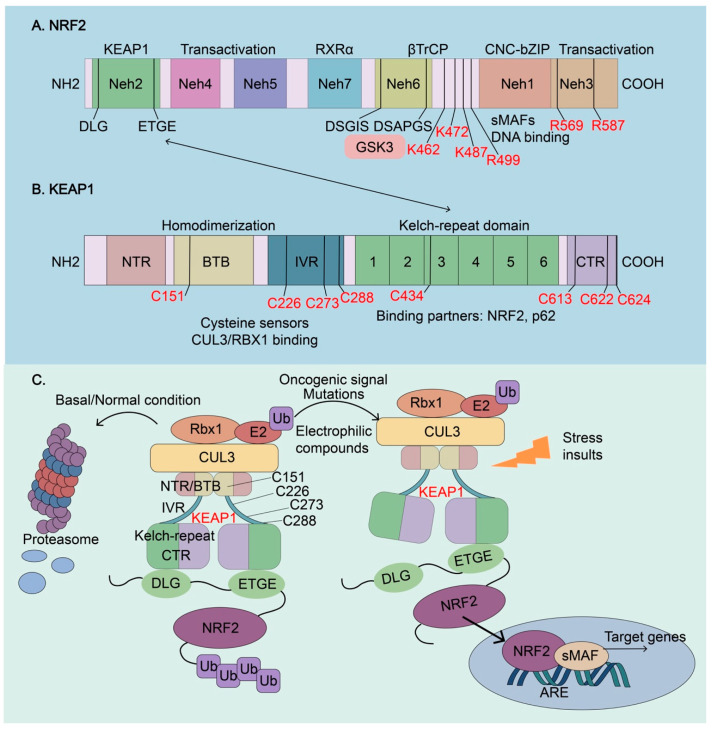
Molecular architectures of NRF2 and KEAP1 and regulation of NRF2 activity. (**A**) The architecture of NRF2. NRF2 has seven conserved NRF2-ECH homology (Neh) domains, Neh1-Neh7. Neh1, Neh4, and Neh5 are transactivation domains, where a basic leucine zipper (bZip) motif is responsible for DNA binding and dimerization with the other transcription cofactors, such as sMAFs. Neh2 contains ETGE and DLG motifs, which are required for the interaction with KEAP1 and subsequent KEAP1-mediated proteasomal degradation. Neh6 contains two βTrCP degrons DSGIS and DSAPGS that are responsible for the β-TrCP mediated proteasomal degradation. Glycogen synthase kinase-3 (GSK3) can phosphorylate the DSGIS motif, resulting in the increased affinity for βTrCP, to enhance NRF2 ubiquitination and degradation. Neh7 interacts with the retinoid X receptor α (RXR), which inhibits NRF2 transcriptional activity. De-glycation of lysines (K462, K472, and K487) and arginines (R499, R569, and R587) in the C-terminus of NRF2, which is mediated by fructosamine-3-kinase, is essential for its stabilization and oncogenic action. (**B**) The architecture of KEAP1. KEAP1 contains five domains, an amino-terminal region (NTR), a broad complex, tram track, bric-à-brac (BTB) domain, an intervening region (IVR), six Kelch-repeat (also named double glycine repeat) domains, and the C-terminal region (CTR). The Kelch domain and CTR mediate the interactions with NRF2 and p62 through their DLG or ETGE motifs. The BTB domain homodimerizes with KEAP1 and contributes to the interaction of IVR with the CUL3/RBX1 complex. Several functional important cysteine residues (C151, C226, C273, C278, C434, C613, C622, and C624), which sense reactive oxygen species (ROS) and electrophiles, modulate KEAP1-NRF2 interaction. (**C**) Regulation of NRF2 activity. Under basal or normal conditions, the continuous sequestration of NRF2 by a KEAP1 dimer through Neh2-Kealch domain interaction results in its subsequent proteasomal degradation and keeps NRF2 activity low. Stressed insults, oncogenic signaling, electrophilic compounds, and activating mutations disrupt the KEAP1-NRF2 complex and lead to the temporary or constitutive increase of cellular NRF2 amount. NRF2 accumulates in the nucleus, where it interacts with other transcription factors and cofactors to regulate the transcription of its target genes. NRF2, Nuclear factor erythroid 2-related factor 2; KEAP1, Kelch-like-ECH-associated protein 1; βTrCP, β-transducing repeat-containing protein; CUL3, Cullin3; RBX1, RING-box protein; RXRα, retinoic X receptor alpha; GSK3, Glycogen synthase kinase-3; SKP1, S-phase kinase-associated protein-1.

The NRF2-KEAP1 system is the primary sensor of oxidative stresses and regulates redox homeostasis. Highly relevant to human disease, NRF2, encoded by the gene nuclear factor, erythroid derived 2 like 2 (*NFE2L2*) and its mouse homolog (*Nfe2l2*), is a master transcription factor for oxidative stress response [43]. NRF2 belongs to the Cap’n’Collar (CNC) basic leucine zipper (bZIP) transcription factor family and *NFE2L2* was first identified by screening the proteins that bind to the tandem consensus sequence for activating protein 1 (AP1) and NF-E2 in the beta-globin locus control region [44]. Subsequent studies revealed that NRF2 is a master regulator of cellular antioxidant stress response and drug detoxification by inducing the expression of many genes that contain an antioxidant response element (ARE) in their promoter regions [5]. Together with the help of other transcription factors and co-factors, such as small musculoaponeurotic fibrosarcoma proteins (sMAFs), NRF2 regulates the transcriptional activation of its target genes [45]. NRF2 itself is regulated by a variety of extracellular and intracellular signals that converge on the nuclear accumulation of NRF2 [45,46]. NRF2 contains seven conserved NRF2-ECH homology (Neh) domains, which determine the complex regulatory network of NRF2 activation (Figure 3A). The Neh1 domain contains a bZIP region, in which the basic region is responsible for DNA binding and ZIP associates with NRF2 dimerization partners, including sMAFs and other bZIP proteins [23]. The Neh2 domain harbors two highly conserved DLG and ETGE motifs that specifically interact with the Kelch-repeated domain of Kelch-like-ECH-associated protein 1 (KEAP1), leading to NRF2 ubiquitylation and subsequent proteasomal degradation [47]. The Neh3-5 domains harbor the transactivation domain and bind to various components of the transcriptional machinery to activate the transcription of NRF2 target genes [45]. The Neh6 domain contains two conserved peptide motifs, DSGIS and DSAPGS that are recognized by beta-transducing repeat-containing protein (βTrCP) to mediate KEAP1-independent NRF2 degradation [48]. In addition, the DSGIS motif can be phosphorylated by glycogen synthase kinase-3 (GSK3), resulting in the increased affinity for βTrCP and enhancing NRF2 ubiquitination and degradation [48,49]. The Neh7 domain interacts with the retinoid X receptor α (RXR) that inhibits NRF2 transcriptional activity [50].

KEAP1 is a redox-regulated adaptor for the Cullin (Cul)3-RING-box protein (Rbx)1 ubiquitin ligase complex [51]. Structurally, KEAP1 contains five domains, an N-terminal domain (NTR), a Broad complex/Tram track/Bric-à-brac (BTB) domain for homodimerization, a central linker intervening region (IVR), a Kelch-repeat domain with 6 Kelch double glycine repeats (DGR), and a C-terminal domain (CTR) (Figure 3B) [52,53]. KEAP1 is one of the best-characterized redox sensors, harboring several sensor cysteines, especially cysteine 151 (C151), C226, C273, C288, C434, C613, and C622/624, which can be oxidized by ROS and other oxidants to sense the oxidative stress [54,55,56,57,58,59]. The H_2_O_2_ sensor of KEAP1 is distinct from that used for sensing electrophiles [56]. Under normal conditions, the ETGE and DLG motifs of NRF2 bind to a KEAP1 dimer [47], and the BTB domain of KEAP1 mediates the binding of NRF2/KEAP1 complex to the CUL3 for NRF2 ubiquitination and degradation (Figure 3C). During oxidative stress, electrophiles and oxidants induce alkylation or oxidation of KEAP1 cysteines and subsequently, conformational changes of KEAP1, resulting in the liberation of NRF2 from KEAP1-E3 ligase complex, disruption of ubiquitylation, and degradation of NRF2 allowing NRF2 translocation to the nucleus to activate the expression of antioxidant genes [54]. Therefore, KEAP1 is the central hub for sensing endogenous and environmental oxidative and electrophilic stress. The resulting newly transcribed and translated NRF2 can accumulate in the nucleus and lead to the activation of cytoprotective and metabolic genes that adapt to the hostile oxidative environment [43]. Many NRF2 activators are direct or indirect KEAP1 cysteine modifiers.

NRF2 is expressed in all cell types and under normal conditions, NRF2 is sequestered in the cytoplasm and targeted for proteasomal degradation mediated by KEAP1 to keep its basal protein level low (Figure 3C) [23,43]. In the presence of ROS and electrophilic compounds, various ROS sensing mechanisms, particularly modification of key cysteine residues in KEPA1 and subsequent conformational change that disrupts its interaction with NRF2, lead to translocation of NRF2 to the nucleus where it promotes transcription of target genes (Figure 3C). Apart from KEAP1 modifications, other posttranslational modifications of NRF2 also control its abundance and activity. De-glycation of lysines (K462, K472, and K487) and arginines (R499, R569, and R587) at its C-terminus, which is mediated by fructosamine-3-kinase, is essential for its stabilization and oncogenic action [60]. In addition, NRF2 can be activated by NRF2-activators or inducers such as sulforaphane, dimethyl fumarate, or bardoxolone, which has been proposed as a treatment strategy for some disorders [46,61]. Somatic mutations in the KEAP1 or NRF2 gene in cancer cells as well as other mutations that disrupt the binding of KEAP1 to NRF2 lead to aberrant NRF2 activation [60,62]. NRF2 has been shown to coordinate with other transcription factors, such as NF-κB, ATF4, MYC, and BMAL1, for the regulation of complex biological processes [46,60,63,64,65]. NRF2 lies at the center of a complex regulatory network of redox homeostasis and cancer development as well as cancer therapy [66].

### 3.2. The Heme Oxygenase (HO) System as Sensor of ROS

The HO system contains the oxidative stress-inducible protein HO-1 and the constitutive isoform HO-2. Using heme, NADPH, and O_2_ as reaction substrates, HO-1 and HO-2 both catalyze the oxidative cleavage of heme at the alpha-methene bridge carbon, released as ferrous iron, biliverdin, and carbon monoxide (CO), a heme ligand. CO is considered a signaling molecule, and biliverdin can be further reduced to potent antioxidant bilirubin catalyzed by biliverdin reductase (BVR) at the expense of NADPH. HO-1 is sensitive to all kinds of stimuli that cause oxidative stress and pathological conditions, including hyperoxia, hypoxia, GSH depletion, heat shock, ischemia, radiation, metal ions, cellular transformations, and disease states. Expression HO-1 is transcriptionally regulated by NRF2 [23]. In contrast, HO-2 has two cysteine residues Cys265 and Cys282 in its heme regulatory motif, which exhibit O_2_ sensitivity. The two cysteine residues are necessary for the activity of HO-2. Hypoxia inhibits HO-2 activity and decreases its product, CO, from the heme oxidative cleavage. CO itself stimulates protein kinase G (PKG)-dependent phosphorylation of Ser377 of cystathionine-γ-lyase (CSE), leading to the inhibition of H_2_S generation by CSE [67]. Therefore, reduced CO levels under hypoxia increase the H_2_S synthesis by CSE and ultimately the carotid body activation for O_2_ homeostasis.

### 3.3. Hypoxia-Inducible Factors (HIFs) as O_2_ Sensors

**Figure 4:**
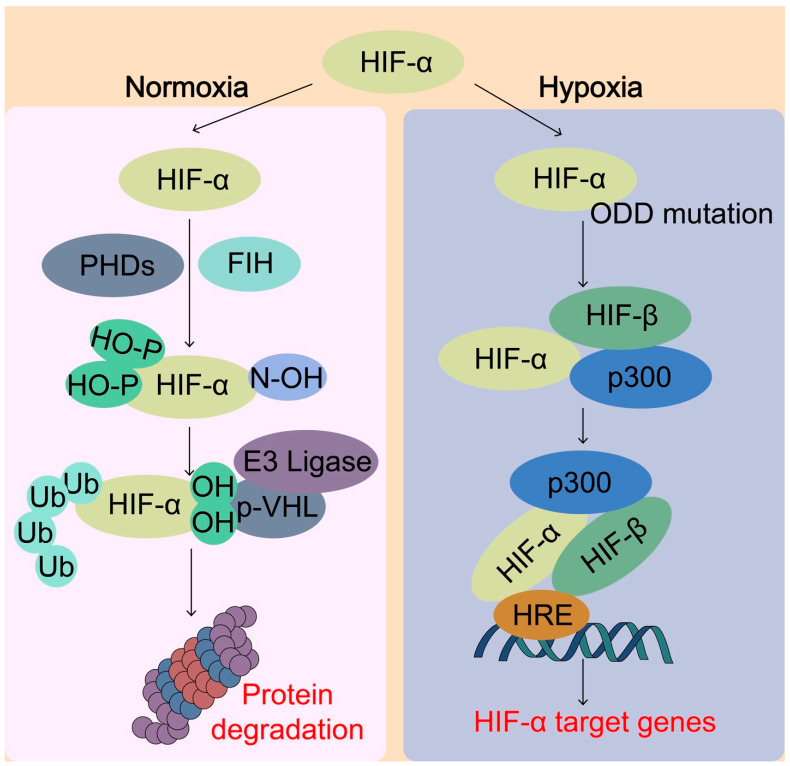
Sensing of O_2_ by hypoxia-inducible factor (HIF) system. Under normal oxygen conditions (normoxia), the HIF-α protein is rapidly hydroxylated by the prolyl hydroxylases (PHDs) enzymes, which enables the binding of von Hippel–Lindau (pVHL) E3 ubiquitin ligase complex, leading to ubiquitination and proteasomal degradation of HIF-α. On the other hand, the factor inhibiting HIF-1 (FIH) catalyzes specific asparagine hydroxylation of HIF-α, which blocks the transcriptional co-activator p300 from binding with HIF-α, thereby inhibiting HIF transcriptional activity. Under hypoxic conditions, the activity of PHDs and FIH is inhibited due to lack of O_2_, thereby lack of hydroxylation of HIF-α. Unhydroxylated HIF-α translocates to the nucleus, forms a complex with HIF-β and p300 targeting hypoxia response element (HRE), and activates transcription of HIF target genes. pVHL, von Hippel-Lindau tumor-suppressor protein; HIF, hypoxia-inducible factor; ODD, oxygen-dependent degradation domain; p300, Histone acetyltransferase p300.

HIFs are a major O_2_ sensing cellular response mechanism that an aerobic organism has developed to maintain O_2_ homeostasis. HIFs are members of the basic helix–loop–helix Per–Arnt–Sim (bHLH-PAS) family of transcription factors that regulate the transcription of thousands of genes [68]. The HIFs function as heterodimeric proteins that contain an O_2_-regulated HIF-α subunit (HIF-1α, HIF-2α, or HIF-3α) and a constitutively expressed HIF-β subunit. The O_2_ sensing ability is executed by two different types of dioxygenases or hydroxylases, including prolyl hydroxylases (PHDs) and an asparaginyl hydroxylase factor inhibiting HIF (FIH-1) (Figure 4) [68]. Under normoxia, PHDs use the O_2_ to hydroxylate the conserved Pro-564 and Pro-402 residues within the oxygen-dependent degradation domain (ODD) of HIF-1α and the prolyl hydroxylation prompts the recognition by the von Hippel–Lindau tumor-suppressor protein (pVHL), which is a part of E3 ubiquitin ligase complex, for the polyubiquitylation and subsequent proteasomal degradation of HIF-α [69]. Under hypoxia or mutation of ODD, PHD-dependent HIF-α hydroxylation is reduced and HIF-α is stabilized to allow its nuclear translocation and heterodimerization with HIF-β, together with the transcriptional co-activator cAMP-response element binding (CREB)-binding protein (CBP) and histone acetyltransferase p300 (p300). Once the HIF transcriptional complex is formed, it can bind to the hypoxia-responsive elements (HRE) in the promoter or enhancer regions of many target genes for their transcriptional activation. FIH-1 hydroxylates Asn-803 within the C-terminal transactivation domain of HIF-1α under normal O_2_ conditions and hydroxylation of HIF-1α blocks the binding of the HIFα subunits with the transcriptional coactivators CBP/p300, thus inhibiting transcriptional activation [70]. Both PHDs and FIH belong to a large family of α-ketoglutarate (αKG)-dependent hydroxylases that requires O_2_, iron, αKG, and ascorbate in some cases to catalyze the incorporation of O_2_ into organic substrates. αKG is a central metabolic hub, participating in the tricarboxylic acid (TCA) cycle and in amino-acid transamination such as glutaminolysis, which replenishes the TCA cycle. By sensing O_2_ levels, HIFs induce the transcription of many target genes involved in the regulation of redox homeostasis, metabolism, and development. 

### 3.4. Other Sensors of ROS and O_2_

Several other PHD substrates have been identified, including transcription factors p53, nuclear factor-κB (NF-κB), and forkhead box O (FOXO) family of transcription factors. Tumor suppressor p53 can be hydroxylated by PHD3 at Pro359, which is involved in the binding of deubiquitinases (DUBs), and increases its stability by enhancing its deubiquitination by DUBs ubiquitin specific peptidase 7/10 (USP7/10) [71]. In addition, p53 can also be modulated by H_2_O_2_ through direct oxidation of p53 cysteine residues and indirect modulation of signaling networks [72]. As a result, p53 regulates cellular redox homeostasis by inducing the expression of antioxidant genes. NF-κB is a master regulator of inflammation and innate immune responses and it regulates the expression of many inflammatory cytokines and chemokines that determine the cancer microenvironment favoring cancer progression and chemoresistance [73,74,75,76]. NF-κB and its activating kinase, IKK, have become appealing therapeutic targets because they are constitutively active in many cancers [77,78]. NF-κB signaling has both anti- and pro-oxidant roles and ROS can both activate and suppress NF-κB function in a phase and context-dependent manner [79]. ROS-mediated Cys179 oxidation and modifications of inhibitor of NF-κB (IκB) kinases (IKK) inhibited IKK activity [79]. In addition, ROS also directly modify Cys62 in the RHD domain of the NF-κB p50 subunit to decrease its DNA binding activity, leading to the reduced transcriptional activity of NF-κB [79]. In addition, NF-κB regulates a new type of pH-dependent form of regulated cell death, named alkaliptosis, by inhibiting carbonic anhydrase 9, a key enzyme regulating pH balance in cells, rendering alkaliptosis as a new strategy for cancer therapy [80]. Generally, NF-kB activity is negatively correlated with NRF2 activity [23,64] and NRF2 activity could be harnessed for the regulation of alkaliptosis in cancer prevention and therapy. Transcription factor FOXO3a can be hydroxylated at Pro426 and Pro437 by PHD1 and FOXO3a hydroxylation blocks its association with deubiquitinase USP9 X-linked (USP9x), resulting in its proteasomal degradation [81]. FOXO transcription factors can transcriptionally repress some of the gene expressions, such as Cyclin D1, and insufficient FOXO3a hydroxylation promotes its accumulation, which in turn suppresses Cyclin D1 expression [81]. The histone lysine demethylases (KDMs), a subfamily of αKG-dependent hydroxylases, are a group of important epigenetic regulatory proteins that shape the chromatin structure and gene transcription, including H3K27 demethylase KDM6A [82] and KDM3A [83]. Because the hydroxylases have a relatively high affinity with O_2_, they often serve as an O_2_ sensor in the process of histone demethylation and hypoxic reprogramming. 

## 4. NRF2 Functions in Carcinogenesis and Cancer Development

### 4.1. NRF2 Regulates Glutathione Metabolism and Antioxidant Defense in Tissue Injury and Tumorigenesis

**Figure 5:**
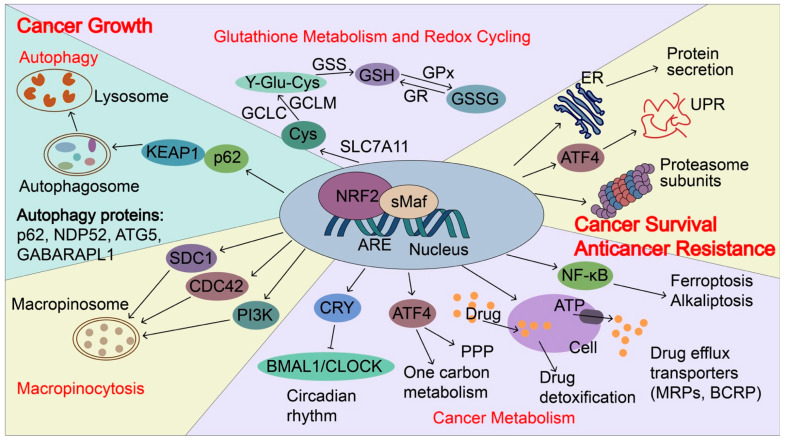
NRF2-induced targets regulate cancer growth, metastasis, and cancer drug resistance. In cancers, NRF2 signaling is aberrantly activated and NRF2 induces p62 expression and autophagy. p62 can directly interact with KEAP1, which causes NRF2 accumulation and KEAP1 degradation via the autophagy-related pathway. NRF2 transactivates the expression of genes encoding antioxidant proteins and drug-metabolizing enzymes, including glutathione metabolism and redox recycling. For example, NRF2 regulates the expression of SLC7A11 (xCT), Glutamate--cysteine ligase (GCLC and GCLM), and glutathione synthetase (GSS), all of which contribute to the elevation of reduced GSH levels. In addition, NRF2 regulates genes involved in autophagy, cancer metabolism, and macropinocytosis to support the nutrients demands for the rapid growth of cancer cells, including p62, antigen nuclear dot 52 kDa protein (NDP52), Autophagy protein 5 (ATG5), Gamma-aminobutyric acid receptor-associated protein-like 1 (GABARPL1), Syndecan-1 (SDC1), Cell division control protein 42 homolog (CDC42) and Phosphatidylinositol 4,5-bisphosphate 3-kinase catalytic subunit gamma isoform (PIK3CG). Furthermore, NRF2 regulates unfolded protein response (UPR), proteostasis, cancer metabolism, and drug detoxification, which confer cancer metastasis and anticancer resistance. NRF2 transactivates the expression of genes encoding proteasome subunits, drug efflux transporters, multidrug resistance-associated proteins (MRPs/ABCCs), and breast cancer resistance proteins (BCRP/ABCG2). NRF2 also increases ATF4 expression and coordinates with ATF4 to regulate cancer metabolism, UPR, and protein secretion, as well as to maintain the state of the drug-tolerant persister cells. Furthermore, NRF2 represses CLOCK/BMAL1-regulated circadian rhythm by inducing the expression of CRY2 and integrating cellular redox signals and metabolism to promote tumorigenesis, cancer growth, and drug resistance. NDP52, antigen nuclear dot 52 kDa protein; ATG5, autophagy protein 5; GABARPL1, gamma-aminobutyric acid receptor-associated protein-like 1; SDC1, syndecan-1; CDC42, cell division control protein 42 homologs; PIK3CG, phosphatidylinositol 4,5-bisphosphate 3-kinase catalytic subunit gamma isoform; BMAL1: brain and muscle ARNT-like protein1; CLOCK: Circadian locomotor output cycles kaput; CRY2: Cryptochrome 2; GSS, glutathione synthetase; MRPs, multidrug resistance-associated proteins; BCRP, breast cancer resistance protein; *SLC7A11*(xCT), Solute carrier family 7 and member 11 (Cystine/glutamate transporter); GCLC, glutamate-cysteine ligase catalytic subunit; GCLM, glutamate-cysteine ligase modulator.

O_2_ supply and demand imbalances cause ROS, and ROS induce the activation of NRF2 that in turn regulates cellular redox balance through the induction of professional enzymes dedicated to preventing the build-up of intracellular ROS, including the enzymes in phase I, II, and III of the drug detoxification reaction and elimination of pro-oxidants to maintain cellular homeostasis (Figure 5) [43]. Glutathione is a major antioxidant and is made from three amino acids: glycine, L-cysteine, and L-glutamate. The intracellular availability of Cys is determined by glutamate–cystine antiporter (xCT), encoded by solute carrier family 7 member 11 (*SLC7A11*), which exports glutamate in exchange for cystine uptake [84,85]. xCT expression is regulated by NRF2 [62]. NRF2 regulates glutathione metabolism through the induction of enzymes in glutathione synthesis, reduction, and redox cycling enzymes. Glutamate-cysteine ligase catalytic (GCLC) and modulator (GCLM) subunits as well as glutathione synthetase (GSS) are the three NRF2 targets involved in the GSH synthesis and NRF2 stimulates the expression of GCLC, GCLM, and GSS (Figure 5) [43,62,85]. NRF2 induces the expression of GR, GPx2, GPx4, SOD1, CAT, and several glutathione S-transferases, which are responsible for glutathione utilization and redox cycling [43]. In addition, ROS can oxidize the Cys of proteins to the sulfenic form, resulting in structural changes of the proteins that alter their functions. The sulfenic form of Cys can be reduced to thiolate anions by the disulfide reductases thioredoxin (TRX) and glutaredoxin (GRX) to return the protein function to its original state [86]. TRX and GRX are direct targets of NRF2 [87]. By decreasing oxidative stress, NRF2 can prevent tissue and cell damage, thereby decreasing inflammation. *Nfe2l2*-deficient mice are also highly susceptible to drug-induced liver injury, alcoholic liver disease, and non-alcoholic fatty liver disease [88]. Compare with wild-type mice, *Nfe2l2*-null mice displayed more severe lung inflammation and damage, contributing to the pathogenesis of emphysema, upon exposure to cigarette smoke [89]. In addition, in a hyperoxia-induced acute lung injury (ALI) model, NRF2-knockout mice exhibited persistent cellular injury, impaired alveolar and endothelial cell regeneration, and persistent cellular infiltration by macrophages and lymphocytes, and this hyperoxia-induced damage was rescued by glutathione supplementation [90]. Considering the protective roles of NRF2 in tissue injury and repair, *Nfe2l2^−/−^* mice are more susceptible to chemical- and radiation-induced tumorigenesis, and NRF2 activators were reported to reduce the burdens of several cancers, including liver cancer [91,92], colon cancer [93], breast cancer [94], prostate cancer [95], and bladder cancer [96]. Therefore, as a master regulator of stress response against oxidative and toxic insults, NRF2 activation suppresses tissue injury, tumor-promoting inflammation, and cancer initiation. 

NRF2-activating mutations and loss of function mutations in KEAP1 and Cul3 that prevent effective NRF2 repression frequently occur in many cancers, such as liver cancer [97], lung cancer [98], ovarian cancer [99], kidney cancer [100], and breast cancer [101], resulting in constitutive activation of NRF2 signaling in cancer cells. All NRF2 mutations are located within the DLG (43%) and ETGE (57%) motifs, which are critical sites for the binding of NRF2 to KEAP1 [43,102]. About 19% of patients with lung cancer harbor somatic mutations in *KEAP1*, the third most commonly mutated gene behind the tumor-suppressor TP53 (46%) and KRAS (32%) oncogene [102,103]. Those mutations leading to NRF2 activation support the broad cancer-promoting roles of NRF2. Recent studies have shown that NRF2 has broader functions in regulating cancer progression. 

### 4.2. NRF2 Regulates Autophagy, Cancer Metabolism, and Macropinocytosis for Cancer Growth

NRF2 regulates autophagy, cancer metabolism, and macropinocytosis to support the nutrient demands for the rapid growth of cancer cells. Autophagy is a vital process in which the body’s cells “clean out” any unnecessary or damaged components to allow intracellular nutrient recycling, especially under starvation conditions. Due to limited nutrient supply, cancer cells often elevate autophagy and depend on autophagy-mediated scavenging and recycling of intracellular macromolecules to maintain survival and growth [63,104]. NRF2 induces the expression of autophagy genes, including SQSTM1/p62, calcium-binding and coiled-coil domain-containing protein 2 (CALCOCO2/NDP52), autophagy protein 5 (ATG5), and gamma-aminobutyric acid receptor-associated protein-like 1 (GABARAPL1) to enhance autophagy [105]. In addition, NRF2 stimulates aerobic glycolysis, pentose phosphate pathway (PPP), de novo purine biosynthesis pathway, and amino acid and one-carbon metabolism to support cancer proliferation. The NRF2-induced genes to reprogram cancer metabolism have been extensively reviewed elsewhere [43,106]. In addition to the autophagy that supports intracellular nutrient recycling, a recent study showed that NRF2 mediates transcription of genes encoding the macropinocytosis pathway components, surface-localized syndecan 1 (SDC1), Na^+^/H^+^ exchanger 1 (NHE1), CDC42, and PIK3CG that induces an alternative route for tumors to scavenge nutrients from extracellular sources [107]. In pancreatic ductal adenocarcinoma (PDAC), NRF2 regulates collagenolysis and enables desmoplastic cancers to escape nutrient limitation, thus influencing patient survival [108]. Therefore, NRF2 provides various pathways for nutrient support to cancer cells and enables the growth advantages of cancer cells. This will make us reconsider the anti-cancer therapy based on blocking cancer nutrient supply, especially in NRF2 highly expressed cancers or cancers with super-activated NRF2 mutations. These NRF2-mediated nutrient support pathways could be promising anticancer targets.

### 4.3. NRF2 Regulates Unfolded Protein Response (UPR) and Proteostasis for Cancer Metastasis and Resistance to Anticancer Therapy

UPR is the mechanism by which cells control endoplasmic reticulum (ER) protein synthesis, folding, modification, and transport of secretory and organelle-bound proteins, as well as their degradation. ER stress during cancer growth, stress-rich microenvironments, such as low pH, hypoxia, nutrition deprivation, and metabolic stress, can induce ROS formation and accumulation of misfolded proteins that lead to ER stress and UPR via activation of three signaling arms coordinated by IRE1-XBP1, PERK-eIF2a-ATF4, and ATF6. During metastasis, metastasizing cancer cells have to be able to survive from migration through the stroma, intravasation through the endothelium into the blood or lymphatic vessels, circulation in the vessels, and subsequently extravasation through the endothelium, and colonization at distant tissues. All of these processes exhibit various levels of mechanical forces, including fluid shear stress, hydrostatic pressure, and tension and compression forces, which trigger stress responses. UPR is upregulated in cancers and UPR coordinates with NRF2 to sustain cancer survival, proliferation, and metastasis [106]. ER stress and UPR can activate NRF2 by PERK-mediated phosphorylation of NRF2 [109]. Upon targeted and conventional cancer therapies, cancer cells often induce stress response to survive the cancer treatment and generate drug tolerance via heme-regulated inhibitor (HRI) kinase-ATF4 signaling [110]. Chronic sublethal stress is a major feature of drug-tolerant persister cells. In addition, high NRF2 activity regulates drug detoxification through the induction of antioxidant proteins and phase two metabolizing enzymes. Drug efflux transporters, such as multidrug resistance-associated proteins (MRPs/ABCCs) and breast cancer resistance protein (BCRP/ABCG2), which facilitate xenobiotic detoxification by preventing the intracellular accumulation of foreign substances, contain the functional AREs in their promoter or enhancer regions and they are direct targets of NRF2 [111]. NRF2 activates the transcription of ATF4 which regulates amino-acid metabolism and anticancer drug resistance [112]. NRF2 activation in mice liver induces the expression of genes involved in the UPR and protein secretion [62].

ER stress and UPR share an intimate connection with proteostasis. NRF2 regulates the activity of the proteasome. NRF2 regulates the expression of the 20S proteasome subunits PSMA1, PSMB3, and PSMB6 and 19S proteasome subunits PSMC1, PSMC3, and PSMD14, as well as a proteasome chaperone-proteasome maturation protein (POMP) [113]. Not all of them harbor the conserved ARE motifs, suggesting that NRF2 may coordinate with other transcription factors to induce the transcription of proteasomes subunits. Proteotoxic stress can activate NRF2 by inactivating ARE-transcriptional repressor BACH1 [114]. Elevated NRF2 activation in cancers treated with proteasome inhibitor bortezomib contributes to the bortezomib resistance [114]. In summary, NRF2 increases UPR and proteasome activity, together with the expression of anti-oxidant and drug detoxification enzymes, contributing to stress adaptation.

### 4.4. NRF2 Regulates Circadian Rhythm to Promote Tumorigenesis and Cancer Growth

The circadian rhythm is a natural, internal process that regulates the sleep–wake cycle and other human activities in a manner of roughly every 24-h period of light and dark on earth. It is driven by the circadian clock, an evolutionarily conserved timekeeping system for numerous biological rhythms that allow organisms to anticipate and adapt their behavior and physiology to predictable changes in their environment [115]. Numerous studies indicate that circadian rhythm disruptions (e.g., jet lag, shift work, sleep disruption, and exposure to light at night) are associated with increased cancer risk [46,116,117,118] and World Health Organization designated circadian disruption as a likely carcinogen [115]. The circadian clock is composed of a core transcription-translation feedback loop, in which transcriptional factors aryl hydrocarbon receptor nuclear translocator-like (ARNTL/BMAL1) and circadian locomotor output cycles kaput (CLOCK) activate the transcription of their own repressors, period 1/2/3 (PER1/2/3) and cryptochrome 1/2 (CRY1/2) that in turn represses CLOCK/BMAL1-regulated E-box transcription [46]. Cellular redox potential, metabolism, and circadian rhythms are closely linked. NRF2 is an important bridge between the molecular clock and metabolism. Wible et al. showed that chemical activation of NRF2 or genetic NRF2 activation induces CRY2 expression by binding to the specific enhancer regions of the *CRY2* gene, resulting in the repression of CLOCK/BMAL1-regulated E-box transcription and alteration of circadian rhythms [119]. *Nfe2l2*-deficient mouse fibroblasts, hepatocytes and liver also altered rhythmicity. These data support that NRF2 links metabolism signals to the ticking of the circadian clock. In addition, NRF2 activation at a circadian time corresponding to the peak generation of endogenous oxidative signals resulted in NRF2-dependent reinforcement of circadian amplitude, suggesting that NRF2 amount and/or timing of expression are important to timekeeping in cells. Furthermore, NRF2 itself is also transcriptionally upregulated by BAML1 [120,121]. Therefore, NRF2 and circadian clock comprise an interlocking negative feedback loop that integrates cellular redox signals and metabolism to promote tumorigenesis, cancer growth and drugs resistance. Small molecules targeting the circadian signaling pathways become a new therapeutic method for cancer treatment due to their close relationships with cancer [46]. Therefore, NRF2-targeted small molecules and circadian modifying agents could be combined to treat cancer with better efficiency in the future.

## 5. Conclusions

O_2_ is one of the defining moments in evolution. Oxidation and reduction processes participate in almost all aspects of life, such as mitochondria oxidative phosphorylation, which is the major source of ROS production. Environmental stress is ubiquitous for all living beings and threatens to disrupt cell functions, causing oxidative stress and ROS. ROS can function as both physiological signaling molecules required for numerous cellular processes. However, abnormal levels of ROS promote disease pathogenesis. Timely induction of oxidative stress response and resolution of stress facilitates the tissue repair and wound healing process and ultimately restores a stress-free state or adapts to the stressed state. The amount and duration of ROS could determine the outcome. HIFs, the HO system, the NRF2-KEAP1 system, and several other transcription factors are the common ROS and O_2_ sensing and response pathways. NRF2, as a master regulator of the oxidative stress response, sits at the center of a complex regulatory network that contributes to the initiation and development of many diseases, particularly cancer. The extent and duration of NRF2 activation determine the beneficial or deleterious effects of NRF2. Generally speaking, NRF2 activation executes a protective role under physiological conditions, but it promotes cancer development, metastasis, and anticancer drug resistance after cancer is established. Recent studies uncovered new roles of NRF2 activation in sustaining cancer cell growth and maintaining the drug-tolerant state, including regulation of autophagy, UPR, micropinocytosis, and metabolic reprogramming. Cancer remains an elusive, highly complex, and deadly disease. More NRF2 functions and the crosstalk of NRF2 and other oncogenic pathways in cancer will be uncovered in the future. Timely activation of NRF2 can be a promising pharmacological target to prevent and treat cancer. Selective NRF2 modulators can be used as adjuvant therapy after conventional chemotherapy, targeted therapy, and immunotherapy.

## Data Availability

All data needed to evaluate the conclusions of the paper are present in the paper.

## References

[1] Harris I.S., DeNicola G.M. (2020). The Complex Interplay between Antioxidants and ROS in Cancer. Trends Cell Biol..

[2] Halliwell B. (2022). Reactive oxygen species (ROS), oxygen radicals and antioxidants: Where are we now, where is the field going and where should we go?. Biochem. Biophys. Res. Commun..

[3] McKeown S.R. (2014). Defining normoxia, physoxia and hypoxia in tumours-implications for treatment response. Br. J. Radiol..

[4] Wei H. (1992). Activation of oncogenes and/or inactivation of anti-oncogenes by reactive oxygen species. Med. Hypotheses.

[5] Tossetta G., Marzioni D. (2022). Natural and synthetic compounds in Ovarian Cancer: A focus on NRF2/KEAP1 pathway. Pharmacol. Res..

[6] Kumar B., Adebayo A.K., Prasad M., Capitano M.L., Wang R., Bhat-Nakshatri P., Anjanappa M., Simpson E., Chen D., Liu Y. (2022). Tumor collection/processing under physioxia uncovers highly relevant signaling networks and drug sensitivity. Sci. Adv..

[7] Aragones J., Fraisl P., Baes M., Carmeliet P. (2009). Oxygen sensors at the crossroad of metabolism. Cell Metab..

[8] Pringle K.G., Kind K.L., Sferruzzi-Perri A.N., Thompson J.G., Roberts C.T. (2010). Beyond oxygen: Complex regulation and activity of hypoxia inducible factors in pregnancy. Hum. Reprod. Update.

[9] Tossetta G., Fantone S., Giannubilo S.R., Marinelli Busilacchi E., Ciavattini A., Castellucci M., Di Simone N., Mattioli-Belmonte M., Marzioni D. (2019). Pre-eclampsia onset and SPARC: A possible involvement in placenta development. J. Cell Physiol..

[10] Emanuelli M., Sartini D., Molinelli E., Campagna R., Pozzi V., Salvolini E., Simonetti O., Campanati A., Offidani A. (2022). The Double-Edged Sword of Oxidative Stress in Skin Damage and Melanoma: From Physiopathology to Therapeutical Approaches. Antioxidants.

[11] Di Meo S., Reed T.T., Venditti P., Victor V.M. (2016). Role of ROS and RNS Sources in Physiological and Pathological Conditions. Oxid. Med. Cell Longev..

[12] Bisbach C.M., Hass D.T., Robbings B.M., Rountree A.M., Sadilek M., Sweet I.R., Hurley J.B. (2020). Succinate Can Shuttle Reducing Power from the Hypoxic Retina to the O2-Rich Pigment Epithelium. Cell Rep..

[13] Spinelli J.B., Rosen P.C., Sprenger H.G., Puszynska A.M., Mann J.L., Roessler J.M., Cangelosi A.L., Henne A., Condon K.J., Zhang T. (2021). Fumarate is a terminal electron acceptor in the mammalian electron transport chain. Science.

[14] Kumar R., Landry A.P., Guha A., Vitvitsky V., Lee H.J., Seike K., Reddy P., Lyssiotis C.A., Banerjee R. (2022). A redox cycle with complex II prioritizes sulfide quinone oxidoreductase-dependent H(2)S oxidation. J. Biol. Chem..

[15] Cecchini G. (2022). Complexities of complex II: Sulfide metabolism in vivo. J. Biol. Chem..

[16] Sahoo S., Meijles D.N., Pagano P.J. (2016). NADPH oxidases: Key modulators in aging and age-related cardiovascular diseases?. Clin. Sci..

[17] Magnani F., Mattevi A. (2019). Structure and mechanisms of ROS generation by NADPH oxidases. Curr. Opin. Struct. Biol..

[18] Magnani F., Nenci S., Millana Fananas E., Ceccon M., Romero E., Fraaije M.W., Mattevi A. (2017). Crystal structures and atomic model of NADPH oxidase. Proc. Natl. Acad. Sci. USA.

[19] Moller M.N., Cuevasanta E., Orrico F., Lopez A.C., Thomson L., Denicola A. (2019). Diffusion and Transport of Reactive Species Across Cell Membranes. Adv. Exp. Med. Biol..

[20] Sies H., Jones D.P. (2020). Reactive oxygen species (ROS) as pleiotropic physiological signalling agents. Nat. Rev. Mol. Cell Biol..

[21] Yoboue E.D., Sitia R., Simmen T. (2018). Redox crosstalk at endoplasmic reticulum (ER) membrane contact sites (MCS) uses toxic waste to deliver messages. Cell Death Dis..

[22] Fridovich I. (1997). Superoxide anion radical (O2-.), superoxide dismutases, and related matters. J. Biol. Chem..

[23] He F., Antonucci L., Karin M. (2020). NRF2 as a regulator of cell metabolism and inflammation in cancer. Carcinogenesis.

[24] Parween F., Gupta R.D. (2022). Insights into the role of paraoxonase 2 in human pathophysiology. J. Biosci..

[25] Shakhparonov M.I., Antipova N.V., Shender V.O., Shnaider P.V., Arapidi G.P., Pestov N.B., Pavlyukov M.S. (2018). Expression and Intracellular Localization of Paraoxonase 2 in Different Types of Malignancies. Acta Nat..

[26] Wang X., Xu G., Zhang J., Wang S., Ji M., Mo L., Zhu M., Li J., Zhou G., Lu J. (2019). The clinical and prognostic significance of paraoxonase-2 in gastric cancer patients: Immunohistochemical analysis. Hum. Cell.

[27] Bacchetti T., Salvolini E., Pompei V., Campagna R., Molinelli E., Brisigotti V., Togni L., Lucarini G., Sartini D., Campanati A. (2021). Paraoxonase-2: A potential biomarker for skin cancer aggressiveness. Eur. J. Clin. Investig..

[28] Nagarajan A., Dogra S.K., Sun L., Gandotra N., Ho T., Cai G., Cline G., Kumar P., Cowles R.A., Wajapeyee N. (2017). Paraoxonase 2 Facilitates Pancreatic Cancer Growth and Metastasis by Stimulating GLUT1-Mediated Glucose Transport. Mol. Cell.

[29] Lennicke C., Cocheme H.M. (2021). Redox metabolism: ROS as specific molecular regulators of cell signaling and function. Mol. Cell.

[30] Stockwell B.R., Friedmann Angeli J.P., Bayir H., Bush A.I., Conrad M., Dixon S.J., Fulda S., Gascon S., Hatzios S.K., Kagan V.E. (2017). Ferroptosis: A Regulated Cell Death Nexus Linking Metabolism, Redox Biology, and Disease. Cell.

[31] Stockwell B.R. (2022). Ferroptosis turns 10: Emerging mechanisms, physiological functions, and therapeutic applications. Cell.

[32] Kannengiesser C., Gerard B., El Benna J., Henri D., Kroviarski Y., Chollet-Martin S., Gougerot-Pocidalo M.A., Elbim C., Grandchamp B. (2008). Molecular epidemiology of chronic granulomatous disease in a series of 80 kindreds: Identification of 31 novel mutations. Hum. Mutat..

[33] Chen Z., Keaney J.F., Schulz E., Levison B., Shan L., Sakuma M., Zhang X., Shi C., Hazen S.L., Simon D.I. (2004). Decreased neointimal formation in Nox2-deficient mice reveals a direct role for NADPH oxidase in the response to arterial injury. Proc. Natl. Acad. Sci. USA.

[34] Barry-Lane P.A., Patterson C., van der Merwe M., Hu Z., Holland S.M., Yeh E.T., Runge M.S. (2001). p47phox is required for atherosclerotic lesion progression in ApoE(-/-) mice. J. Clin. Investig..

[35] Lan T., Kisseleva T., Brenner D.A. (2015). Deficiency of NOX1 or NOX4 Prevents Liver Inflammation and Fibrosis in Mice through Inhibition of Hepatic Stellate Cell Activation. PLoS ONE.

[36] Liang S., Ma H.Y., Zhong Z., Dhar D., Liu X., Xu J., Koyama Y., Nishio T., Karin D., Karin G. (2019). NADPH Oxidase 1 in Liver Macrophages Promotes Inflammation and Tumor Development in Mice. Gastroenterology.

[37] Matsumoto M., Zhang J., Zhang X., Liu J., Jiang J.X., Yamaguchi K., Taruno A., Katsuyama M., Iwata K., Ibi M. (2018). The NOX1 isoform of NADPH oxidase is involved in dysfunction of liver sinusoids in nonalcoholic fatty liver disease. Free Radic. Biol. Med..

[38] Cui W., Matsuno K., Iwata K., Ibi M., Matsumoto M., Zhang J., Zhu K., Katsuyama M., Torok N.J., Yabe-Nishimura C. (2011). NOX1/nicotinamide adenine dinucleotide phosphate, reduced form (NADPH) oxidase promotes proliferation of stellate cells and aggravates liver fibrosis induced by bile duct ligation. Hepatology.

[39] Sinenko S.A., Starkova T.Y., Kuzmin A.A., Tomilin A.N. (2021). Physiological Signaling Functions of Reactive Oxygen Species in Stem Cells: From Flies to Man. Front. Cell Dev. Biol..

[40] Kumar P., Prabhakar N.R. (2012). Peripheral chemoreceptors: Function and plasticity of the carotid body. Compr. Physiol..

[41] Prabhakar N.R., Semenza G.L. (2015). Oxygen Sensing and Homeostasis. Physiology.

[42] Sin S.Q., Mohan C.D., Goh R.M.W., You M., Nayak S.C., Chen L., Sethi G., Rangappa K.S., Wang L. (2022). Hypoxia signaling in hepatocellular carcinoma: Challenges and therapeutic opportunities. Cancer Metastasis Rev..

[43] He F., Ru X., Wen T. (2020). NRF2, a Transcription Factor for Stress Response and Beyond. Int. J. Mol. Sci..

[44] Moi P., Chan K., Asunis I., Cao A., Kan Y.W. (1994). Isolation of NF-E2-related factor 2 (Nrf2), a NF-E2-like basic leucine zipper transcriptional activator that binds to the tandem NF-E2/AP1 repeat of the beta-globin locus control region. Proc. Natl. Acad. Sci. USA.

[45] Tonelli C., Chio I.I.C., Tuveson D.A. (2018). Transcriptional Regulation by Nrf2. Antioxid. Redox Signal..

[46] Wang Y., Guo H., He F. (2022). Circadian disruption: From mouse models to molecular mechanisms and cancer therapeutic targets. Cancer Metastasis Rev..

[47] Tong K.I., Katoh Y., Kusunoki H., Itoh K., Tanaka T., Yamamoto M. (2006). Keap1 recruits Neh2 through binding to ETGE and DLG motifs: Characterization of the two-site molecular recognition model. Mol. Cell Biol..

[48] Chowdhry S., Zhang Y., McMahon M., Sutherland C., Cuadrado A., Hayes J.D. (2013). Nrf2 is controlled by two distinct beta-TrCP recognition motifs in its Neh6 domain, one of which can be modulated by GSK-3 activity. Oncogene.

[49] Hayes J.D., Chowdhry S., Dinkova-Kostova A.T., Sutherland C. (2015). Dual regulation of transcription factor Nrf2 by Keap1 and by the combined actions of beta-TrCP and GSK-3. Biochem. Soc. Trans..

[50] Wang H., Liu K., Geng M., Gao P., Wu X., Hai Y., Li Y., Li Y., Luo L., Hayes J.D. (2013). RXRalpha inhibits the NRF2-ARE signaling pathway through a direct interaction with the Neh7 domain of NRF2. Cancer Res..

[51] Sihvola V., Levonen A.L. (2017). Keap1 as the redox sensor of the antioxidant response. Arch. Biochem. Biophys..

[52] Dinkova-Kostova A.T., Kostov R.V., Canning P. (2017). Keap1, the cysteine-based mammalian intracellular sensor for electrophiles and oxidants. Arch. Biochem. Biophys..

[53] Horie Y., Suzuki T., Inoue J., Iso T., Wells G., Moore T.W., Mizushima T., Dinkova-Kostova A.T., Kasai T., Kamei T. (2021). Molecular basis for the disruption of Keap1-Nrf2 interaction via Hinge & Latch mechanism. Commun. Biol..

[54] Canning P., Sorrell F.J., Bullock A.N. (2015). Structural basis of Keap1 interactions with Nrf2. Free Radic. Biol. Med..

[55] McMahon M., Lamont D.J., Beattie K.A., Hayes J.D. (2010). Keap1 perceives stress via three sensors for the endogenous signaling molecules nitric oxide, zinc, and alkenals. Proc. Natl. Acad. Sci. USA.

[56] Suzuki T., Muramatsu A., Saito R., Iso T., Shibata T., Kuwata K., Kawaguchi S.I., Iwawaki T., Adachi S., Suda H. (2019). Molecular Mechanism of Cellular Oxidative Stress Sensing by Keap1. Cell Rep..

[57] Cuadrado A., Rojo A.I., Wells G., Hayes J.D., Cousin S.P., Rumsey W.L., Attucks O.C., Franklin S., Levonen A.L., Kensler T.W. (2019). Therapeutic targeting of the NRF2 and KEAP1 partnership in chronic diseases. Nat. Rev. Drug Discov..

[58] Hirose W., Oshikiri H., Taguchi K., Yamamoto M. (2022). The KEAP1-NRF2 System and Esophageal Cancer. Cancers.

[59] Hu C., Eggler A.L., Mesecar A.D., van Breemen R.B. (2011). Modification of keap1 cysteine residues by sulforaphane. Chem. Res. Toxicol..

[60] Sanghvi V.R., Leibold J., Mina M., Mohan P., Berishaj M., Li Z., Miele M.M., Lailler N., Zhao C., de Stanchina E. (2019). The Oncogenic Action of NRF2 Depends on De-glycation by Fructosamine-3-Kinase. Cell.

[61] Szczesny-Malysiak E., Stojak M., Campagna R., Grosicki M., Jamrozik M., Kaczara P., Chlopicki S. (2020). Bardoxolone Methyl Displays Detrimental Effects on Endothelial Bioenergetics, Suppresses Endothelial ET-1 Release, and Increases Endothelial Permeability in Human Microvascular Endothelium. Oxid. Med. Cell Longev..

[62] He F., Antonucci L., Yamachika S., Zhang Z., Taniguchi K., Umemura A., Hatzivassiliou G., Roose-Girma M., Reina-Campos M., Duran A. (2020). NRF2 activates growth factor genes and downstream AKT signaling to induce mouse and human hepatomegaly. J. Hepatol..

[63] Umemura A., He F., Taniguchi K., Nakagawa H., Yamachika S., Font-Burgada J., Zhong Z., Subramaniam S., Raghunandan S., Duran A. (2016). p62, Upregulated during Preneoplasia, Induces Hepatocellular Carcinogenesis by Maintaining Survival of Stressed HCC-Initiating Cells. Cancer Cell.

[64] Khan M.A., Rabbani G., Aggarawal J., Ahmed R.S. (2023). Divulging the Intricacies of Crosstalk Between NF-kB and Nrf-2/Keap1 Pathway in the Treatment of Arthritis by Dimethyl Fumarate. Appl. Biochem. Biotechnol..

[65] Sarcinelli C., Dragic H., Piecyk M., Barbet V., Duret C., Barthelaix A., Ferraro-Peyret C., Fauvre J., Renno T., Chaveroux C. (2020). ATF4-Dependent NRF2 Transcriptional Regulation Promotes Antioxidant Protection during Endoplasmic Reticulum Stress. Cancers.

[66] Kensler T.W., Wakabayashi N. (2010). Nrf2: Friend or foe for chemoprevention?. Carcinogenesis.

[67] Yuan G., Vasavda C., Peng Y.J., Makarenko V.V., Raghuraman G., Nanduri J., Gadalla M.M., Semenza G.L., Kumar G.K., Snyder S.H. (2015). Protein kinase G-regulated production of H2S governs oxygen sensing. Sci. Signal..

[68] Semenza G.L. (2012). Hypoxia-inducible factors in physiology and medicine. Cell.

[69] Ivan M., Kaelin W.G. (2017). The EGLN-HIF O2-Sensing System: Multiple Inputs and Feedbacks. Mol. Cell.

[70] Zhang N., Fu Z., Linke S., Chicher J., Gorman J.J., Visk D., Haddad G.G., Poellinger L., Peet D.J., Powell F. (2010). The asparaginyl hydroxylase factor inhibiting HIF-1alpha is an essential regulator of metabolism. Cell Metab..

[71] Rodriguez J., Herrero A., Li S., Rauch N., Quintanilla A., Wynne K., Krstic A., Acosta J.C., Taylor C., Schlisio S. (2018). PHD3 Regulates p53 Protein Stability by Hydroxylating Proline 359. Cell Rep..

[72] Liu B., Chen Y., St Clair D.K. (2008). ROS and p53: A versatile partnership. Free Radic. Biol. Med..

[73] Tossetta G. (2022). Metformin Improves Ovarian Cancer Sensitivity to Paclitaxel and Platinum-Based Drugs: A Review of In Vitro Findings. Int. J. Mol. Sci.

[74] Zhong Z., Umemura A., Sanchez-Lopez E., Liang S., Shalapour S., Wong J., He F., Boassa D., Perkins G., Ali S.R. (2016). NF-kappaB Restricts Inflammasome Activation via Elimination of Damaged Mitochondria. Cell.

[75] Jia F., Yu Q., Wang R., Zhao L., Yuan F., Guo H., Shen Y., He F. (2023). Optimized Antimicrobial Peptide Jelleine-I Derivative Br-J-I Inhibits Fusobacterium Nucleatum to Suppress Colorectal Cancer Progression. Int. J. Mol. Sci..

[76] Jia F., Yu Q., Zhao L., Shen Y., Guo H., He F. (2022). Sodium New Houttuyfonate Inhibits Cancer-Promoting Fusobacterium nucleatum (Fn) to Reduce Colorectal Cancer Progression. Cancers.

[77] Suhail M., Tarique M., Muhammad N., Naz H., Hafeez A., Zughaibi T.A., Kamal M.A., Rehan M. (2021). A Critical Transcription Factor NF-kappaB as a Cancer Therapeutic Target and its Inhibitors as Cancer Treatment Options. Curr. Med. Chem..

[78] Zhu S., Liu J., Kang R., Yang M., Tang D. (2021). Targeting NF-kappaB-dependent alkaliptosis for the treatment of venetoclax-resistant acute myeloid leukemia cells. Biochem. Biophys. Res. Commun..

[79] Lingappan K. (2018). NF-kappaB in Oxidative Stress. Curr. Opin. Toxicol..

[80] Song X., Zhu S., Xie Y., Liu J., Sun L., Zeng D., Wang P., Ma X., Kroemer G., Bartlett D.L. (2018). JTC801 Induces pH-dependent Death Specifically in Cancer Cells and Slows Growth of Tumors in Mice. Gastroenterology.

[81] Zheng X., Zhai B., Koivunen P., Shin S.J., Lu G., Liu J., Geisen C., Chakraborty A.A., Moslehi J.J., Smalley D.M. (2014). Prolyl hydroxylation by EglN2 destabilizes FOXO3a by blocking its interaction with the USP9x deubiquitinase. Genes Dev..

[82] Chakraborty A.A., Laukka T., Myllykoski M., Ringel A.E., Booker M.A., Tolstorukov M.Y., Meng Y.J., Meier S.R., Jennings R.B., Creech A.L. (2019). Histone demethylase KDM6A directly senses oxygen to control chromatin and cell fate. Science.

[83] Qian X., Li X., Shi Z., Bai X., Xia Y., Zheng Y., Xu D., Chen F., You Y., Fang J. (2019). KDM3A Senses Oxygen Availability to Regulate PGC-1alpha-Mediated Mitochondrial Biogenesis. Mol. Cell.

[84] Sayin V.I., LeBoeuf S.E., Singh S.X., Davidson S.M., Biancur D., Guzelhan B.S., Alvarez S.W., Wu W.L., Karakousi T.R., Zavitsanou A.M. (2017). Activation of the NRF2 antioxidant program generates an imbalance in central carbon metabolism in cancer. Elife.

[85] Fu J., Xiong Z., Huang C., Li J., Yang W., Han Y., Paiboonrungruan C., Major M.B., Chen K.N., Kang X. (2019). Hyperactivity of the transcription factor Nrf2 causes metabolic reprogramming in mouse esophagus. J. Biol. Chem..

[86] Schieber M., Chandel N.S. (2014). ROS function in redox signaling and oxidative stress. Curr. Biol..

[87] Jaganjac M., Milkovic L., Sunjic S.B., Zarkovic N. (2020). The NRF2, Thioredoxin, and Glutathione System in Tumorigenesis and Anticancer Therapies. Antioxidants.

[88] Tang W., Jiang Y.F., Ponnusamy M., Diallo M. (2014). Role of Nrf2 in chronic liver disease. World J. Gastroenterol..

[89] Iizuka T., Ishii Y., Itoh K., Kiwamoto T., Kimura T., Matsuno Y., Morishima Y., Hegab A.E., Homma S., Nomura A. (2005). Nrf2-deficient mice are highly susceptible to cigarette smoke-induced emphysema. Genes Cells.

[90] Reddy N.M., Kleeberger S.R., Kensler T.W., Yamamoto M., Hassoun P.M., Reddy S.P. (2009). Disruption of Nrf2 impairs the resolution of hyperoxia-induced acute lung injury and inflammation in mice. J. Immunol..

[91] Johnson N.M., Egner P.A., Baxter V.K., Sporn M.B., Wible R.S., Sutter T.R., Groopman J.D., Kensler T.W., Roebuck B.D. (2014). Complete protection against aflatoxin B(1)-induced liver cancer with a triterpenoid: DNA adduct dosimetry, molecular signature, and genotoxicity threshold. Cancer Prev. Res..

[92] Kitamura Y., Umemura T., Kanki K., Kodama Y., Kitamoto S., Saito K., Itoh K., Yamamoto M., Masegi T., Nishikawa A. (2007). Increased susceptibility to hepatocarcinogenicity of Nrf2-deficient mice exposed to 2-amino-3-methylimidazo[4,5-f]quinoline. Cancer Sci..

[93] Long M., Tao S., Rojo de la Vega M., Jiang T., Wen Q., Park S.L., Zhang D.D., Wondrak G.T. (2015). Nrf2-dependent suppression of azoxymethane/dextran sulfate sodium-induced colon carcinogenesis by the cinnamon-derived dietary factor cinnamaldehyde. Cancer Prev. Res..

[94] Kim E.H., Deng C., Sporn M.B., Royce D.B., Risingsong R., Williams C.R., Liby K.T. (2012). CDDO-methyl ester delays breast cancer development in BRCA1-mutated mice. Cancer Prev. Res..

[95] Frohlich D.A., McCabe M.T., Arnold R.S., Day M.L. (2008). The role of Nrf2 in increased reactive oxygen species and DNA damage in prostate tumorigenesis. Oncogene.

[96] Iida K., Itoh K., Maher J.M., Kumagai Y., Oyasu R., Mori Y., Shimazui T., Akaza H., Yamamoto M. (2007). Nrf2 and p53 cooperatively protect against BBN-induced urinary bladder carcinogenesis. Carcinogenesis.

[97] Eichenmuller M., Trippel F., Kreuder M., Beck A., Schwarzmayr T., Haberle B., Cairo S., Leuschner I., von Schweinitz D., Strom T.M. (2014). The genomic landscape of hepatoblastoma and their progenies with HCC-like features. J. Hepatol..

[98] Padmanabhan B., Tong K.I., Ohta T., Nakamura Y., Scharlock M., Ohtsuji M., Kang M.I., Kobayashi A., Yokoyama S., Yamamoto M. (2006). Structural basis for defects of Keap1 activity provoked by its point mutations in lung cancer. Mol. Cell.

[99] Konstantinopoulos P.A., Spentzos D., Fountzilas E., Francoeur N., Sanisetty S., Grammatikos A.P., Hecht J.L., Cannistra S.A. (2011). Keap1 mutations and Nrf2 pathway activation in epithelial ovarian cancer. Cancer Res..

[100] Ooi A., Dykema K., Ansari A., Petillo D., Snider J., Kahnoski R., Anema J., Craig D., Carpten J., Teh B.T. (2013). CUL3 and NRF2 mutations confer an NRF2 activation phenotype in a sporadic form of papillary renal cell carcinoma. Cancer Res..

[101] Nioi P., Nguyen T. (2007). A mutation of Keap1 found in breast cancer impairs its ability to repress Nrf2 activity. Biochem. Biophys. Res. Commun..

[102] Campbell J.D., Alexandrov A., Kim J., Wala J., Berger A.H., Pedamallu C.S., Shukla S.A., Guo G., Brooks A.N., Murray B.A. (2016). Distinct patterns of somatic genome alterations in lung adenocarcinomas and squamous cell carcinomas. Nat. Genet..

[103] Cancer Genome Atlas Research N. (2014). Comprehensive molecular profiling of lung adenocarcinoma. Nature.

[104] Taniguchi K., Yamachika S., He F., Karin M. (2016). p62/SQSTM1-Dr. Jekyll and Mr. Hyde that prevents oxidative stress but promotes liver cancer. FEBS Lett..

[105] Pajares M., Jimenez-Moreno N., Garcia-Yague A.J., Escoll M., de Ceballos M.L., Van Leuven F., Rabano A., Yamamoto M., Rojo A.I., Cuadrado A. (2016). Transcription factor NFE2L2/NRF2 is a regulator of macroautophagy genes. Autophagy.

[106] Rojo de la Vega M., Chapman E., Zhang D.D. (2018). NRF2 and the Hallmarks of Cancer. Cancer Cell.

[107] Su H., Yang F., Fu R., Li X., French R., Mose E., Pu X., Trinh B., Kumar A., Liu J. (2021). Cancer cells escape autophagy inhibition via NRF2-induced macropinocytosis. Cancer Cell.

[108] Su H., Yang F., Fu R., Trinh B., Sun N., Liu J., Kumar A., Baglieri J., Siruno J., Le M. (2022). Collagenolysis-dependent DDR1 signalling dictates pancreatic cancer outcome. Nature.

[109] Cullinan S.B., Zhang D., Hannink M., Arvisais E., Kaufman R.J., Diehl J.A. (2003). Nrf2 is a direct PERK substrate and effector of PERK-dependent cell survival. Mol. Cell Biol..

[110] Kalkavan H., Chen M.J., Crawford J.C., Quarato G., Fitzgerald P., Tait S.W.G., Goding C.R., Green D.R. (2022). Sublethal cytochrome c release generates drug-tolerant persister cells. Cell.

[111] Wu K.C., Cui J.Y., Klaassen C.D. (2012). Effect of Graded Nrf2 Activation on Phase-I and -II Drug Metabolizing Enzymes and Transporters in Mouse Liver. PLoS ONE.

[112] DeNicola G.M., Chen P.H., Mullarky E., Sudderth J.A., Hu Z., Wu D., Tang H., Xie Y., Asara J.M., Huffman K.E. (2015). NRF2 regulates serine biosynthesis in non-small cell lung cancer. Nat. Genet..

[113] Kwak M.K., Wakabayashi N., Greenlaw J.L., Yamamoto M., Kensler T.W. (2003). Antioxidants enhance mammalian proteasome expression through the Keap1-Nrf2 signaling pathway. Mol. Cell Biol..

[114] Rushworth S.A., Bowles K.M., MacEwan D.J. (2011). High basal nuclear levels of Nrf2 in acute myeloid leukemia reduces sensitivity to proteasome inhibitors. Cancer Res..

[115] Shafi A.A., Knudsen K.E. (2019). Cancer and the Circadian Clock. Cancer Res..

[116] Wendeu-Foyet M.G., Menegaux F. (2017). Circadian Disruption and Prostate Cancer Risk: An Updated Review of Epidemiological Evidences. Cancer Epidemiol. Biomark. Prev..

[117] Ruan W., Yuan X., Eltzschig H.K. (2021). Circadian rhythm as a therapeutic target. Nat. Rev. Drug Discov..

[118] Kettner N.M., Voicu H., Finegold M.J., Coarfa C., Sreekumar A., Putluri N., Katchy C.A., Lee C., Moore D.D., Fu L. (2016). Circadian Homeostasis of Liver Metabolism Suppresses Hepatocarcinogenesis. Cancer Cell.

[119] Wible R.S., Ramanathan C., Sutter C.H., Olesen K.M., Kensler T.W., Liu A.C., Sutter T.R. (2018). NRF2 regulates core and stabilizing circadian clock loops, coupling redox and timekeeping in Mus musculus. Elife.

[120] Early J.O., Menon D., Wyse C.A., Cervantes-Silva M.P., Zaslona Z., Carroll R.G., Palsson-McDermott E.M., Angiari S., Ryan D.G., Corcoran S.E. (2018). Circadian clock protein BMAL1 regulates IL-1beta in macrophages via NRF2. Proc. Natl. Acad. Sci. USA.

[121] Sun Q., Zeng C., Du L., Dong C. (2021). Mechanism of circadian regulation of the NRF2/ARE pathway in renal ischemia-reperfusion. Exp. Ther. Med..

